# Assessing basic life support skills without an instructor: is it possible?

**DOI:** 10.1186/1472-6920-12-58

**Published:** 2012-07-23

**Authors:** Nicolas Mpotos, Bram De Wever, Martin A Valcke, Koenraad G Monsieurs

**Affiliations:** 1 Emergency Department, Ghent University Hospital, De Pintelaan 185, Ghent, B-9000, Belgium; 2 Department of Educational Studies, Ghent University, H. Dunantlaan 2, Ghent, B-9000, Belgium; 3 Faculty of Medicine and Health Sciences, Ghent University, De Pintelaan 185, Ghent, B-9000, Belgium; 4 Emergency Department, Antwerp University Hospital, Wilrijkstraat 10, Edegem, B-2650, Belgium; 5 Faculty of Medicine and Health Sciences, University of Antwerp, Universiteitsplein 1, Wilrijk, B-2610, Belgium

**Keywords:** Automated testing, Basic Life Support, Cardiopulmonary resuscitation, Self-directed learning

## Abstract

**Background:**

Current methods to assess Basic Life Support skills (BLS; chest compressions and ventilations) require the presence of an instructor. This is time-consuming and comports instructor bias. Since BLS skills testing is a routine activity, it is potentially suitable for automation. We developed a fully automated BLS testing station without instructor by using innovative software linked to a training manikin. The goal of our study was to investigate the feasibility of adequate testing (effectiveness) within the shortest period of time (efficiency).

**Methods:**

As part of a randomised controlled trial investigating different compression depth training strategies, 184 medicine students received an individual appointment for a retention test six months after training. An interactive Flash^TM^ (Adobe Systems Inc., USA) user interface was developed, to guide the students through the testing procedure after login, while Skills Station^TM^ software (Laerdal Medical, Norway) automatically recorded compressions and ventilations and their duration (“time on task”). In a subgroup of 29 students the room entrance and exit time was registered to assess efficiency. To obtain a qualitative insight of the effectiveness, student’s perceptions about the instructional organisation and about the usability of the fully automated testing station were surveyed.

**Results:**

During testing there was incomplete data registration in two students and one student performed compressions only. The average time on task for the remaining 181 students was three minutes (SD 0.5). In the subgroup, the average overall time spent in the testing station was 7.5 minutes (SD 1.4). Mean scores were 5.3/6 (SD 0.5, range 4.0-6.0) for instructional organisation and 5.0/6 (SD 0.61, range 3.1-6.0) for usability. Students highly appreciated the automated testing procedure.

**Conclusions:**

Our automated testing station was an effective and efficient method to assess BLS skills in medicine students. Instructional organisation and usability were judged to be very good. This method enables future formative assessment and certification procedures to be carried out without instructor involvement.

**Trial registration:**

B67020097543

## Background

Delivery of high quality chest compressions is the Basic Life Support (BLS) skill most likely to improve survival [1-5]. To ensure trainees reliably achieve the learning objectives, educational interventions should be evaluated through assessment [6]. Since BLS skills mastery rapidly decays and should not be assumed to persist for pre-defined time periods, regular skill assessment should be established to determine the need for refresher training [6]. Current BLS testing methods require the presence of an instructor, making testing time-consuming with a risk of instructor bias [7]. Acquiring objective data from recording manikins provides more accurate information about skills mastery than instructor judgement. However, current manikin-based solutions still require an instructor to organise testing, to manage the candidates, to present a scenario (when required) and to operate the manikin and the computer.

The goal of our study was to investigate the feasibility of adequate testing (effectiveness) within the shortest period of time (efficiency), using an automated testing procedure without an instructor.

To determine the effectiveness of the testing procedure, we surveyed the participants’ perceptions regarding the key elements in the instructional setting of the automated testing station (goals, instructions, assessment and feedback) and elements related to the setup. In the literature, the latter is labelled as "usability" [8].

Efficiency was measured by a research collaborator who registered the overall time spent in the testing station in a subgroup of students

## Methods

During the academic year 2009–2010, as part of a randomised controlled trial investigating different compression depth training strategies in a self-learning (SL) station, 184 third year medicine students had to be assessed six months after initial training [9]. In order to facilitate the assessment procedure, our objective was to develop a fully automated testing method without instructor and to evaluate if such a method would be able to achieve adequate testing (effectiveness) within the shortest period of time (efficiency). The Ethics Committee of Ghent University Hospital approved the study on 8 December 2009 (trial registration B67020097543). Participation in the study was voluntary, non-participation did not influence student grades. All students had received instructor-led (IL) training and testing during the first and second year of medicine.

The actual testing results are reported in the randomised controlled trial by Mpotos and colleagues [9]. To ensure BLS competency of every student in accordance to the resuscitation guidelines a refresher training was provided in the following year.

### Research procedure

**Figure 1:**
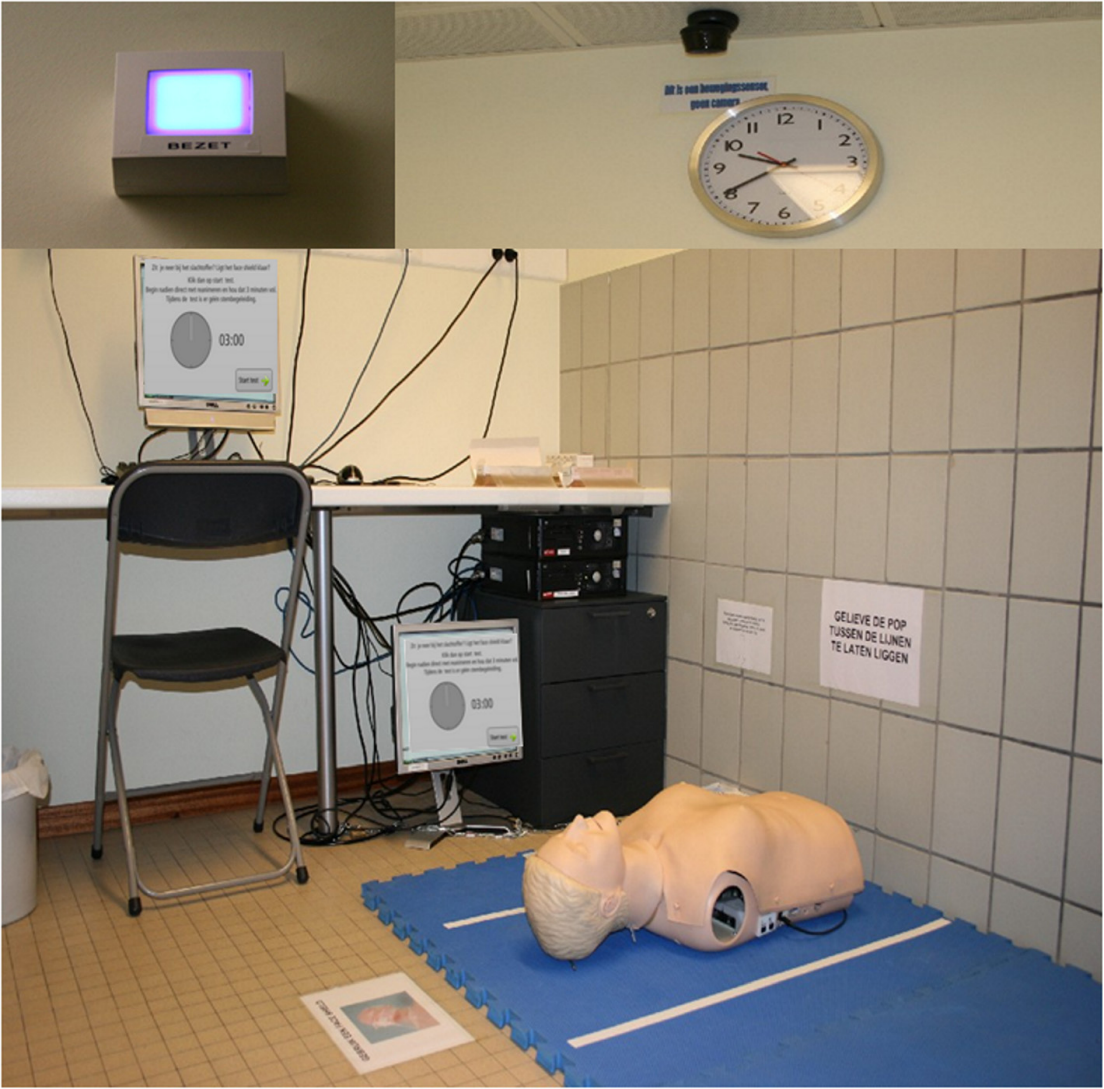
**Testing room equipped with a computer and manikin.** Left upper corner: indicator light outside the room. Right upper corner: clock and infrared sensor to detect presence of a student.

**Figure 2:**
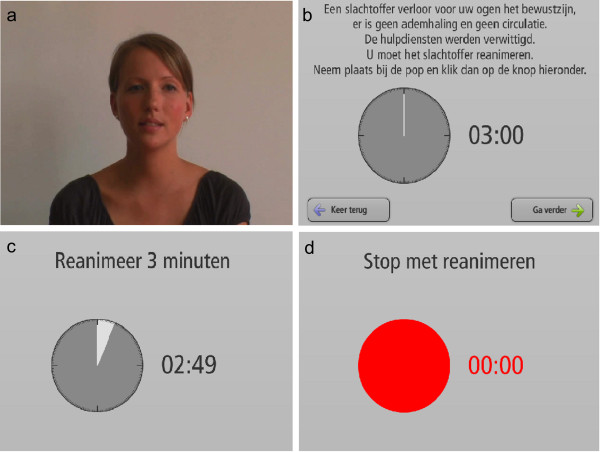
**Screenshots of the Flash module.** Translation of the text is described in the methods section.

We designed an interactive user interface with Flash^TM^ (Adobe Systems Inc., USA) to guide the students through the testing procedure without the presence of an instructor, allowing them to perform BLS skills on a commercially available “Resusci Anne torso” manikin (Laerdal Medical, Norway) during three minutes, while their performance was automatically registered by existing software (Resusci Anne Skills Station^TM^ software version 2.0, Laerdal Medical, Norway) running on a computer. To embed the newly developed Flash^TM^-based user interface in the Resusci Anne Skills Station^TM^ software, Laerdal Medical provided us with a modified version of their commercial software. Laerdal Medical was not further involved in the development of the interactive Flash^TM^ video and the concept of removing the instructor from the testing procedure which was developed at Ghent University.The computer and the manikin were placed in a small room, accessible 24 hours a day, and seven days a week. The room was equipped with an infrared detector connected to an indicator light placed on the outside, notifying when the testing station was in use [Figure 1]. After entering the room and logging in to the computer, a short video message showed an instructor with the following message in the participants’ native language: “Welcome to the resuscitation self-learning station [Figure 2a]. You will be asked to perform a test in relation to your previously acquired resuscitation skills. The test consists of performing BLS for three minutes. For reasons of hygiene the use of a face shield while ventilating the manikin is mandatory. When you feel ready to begin, click the “start test” button. The test cannot be interrupted and will automatically stop after 3 minutes”.

After this introduction, a text message was displayed asking the student to take a face shield and place it on the manikin. By clicking “continue”, the next screen informed the student that a victim had just collapsed in the room, that there was no breathing and circulation and that an ambulance was called. The student was asked to kneel down next to the victim and to resuscitate the victim. The same screen showed an analogue clock and a digital countdown timer of three minutes [Figure 2b]. By clicking “continue”, the next screen asked the student to confirm that he was sitting next to the manikin and that the face shield was properly placed on the manikin’s face. The student was asked to click the “start test” button displayed on the screen and to perform BLS for three minutes [Figure 2c]. Because during training in the SL station six months before the test, students had received automated voice feedback from the manikin, we stressed that the test would be without voice assistance. The Resusci Anne Skills Station^TM^ automatically registered the data picked up by sensors in the manikin. The amount of time spent performing compressions and ventilations was also registered and will be referred to as “time on task”. For our test, a time on task of three minutes was required. Exactly after three minutes, the clock and numeric countdown turned red and an audio warning signal was played. This was immediately followed by a video message from the instructor to announce the end of the test, also asking the student to clean the manikin and to leave the room [Figure 2d]. The program then automatically returned to the login screen and performance results were stored as xml files in a database. Students did not receive feedback about their performance at the end of the test.

Typical information stored in the xml files is illustrated in Table 1. Settings/limits for the different BLS parameters could be modified in a configuration file that was part of the Skills Station^TM^ software.

**Table 1:** Information stored in the xml files

·	program version
·	date
·	login name
·	scenario type
·	total number of compression
·	average compression depth
·	number registered with incomplete release (≥5 mm)
·	number registered with hand position too low/too high up/too far to the right/too far too the left
·	number registered with incorrect hand placement
·	number registered with average, adequate, insufficient and excessive rate, time-outs, total number of ventilations
·	number of ventilations registered with average, adequate, insufficient and excessive volume
·	average minute volume
·	number of ventilations registered with insufficient relaxation
·	average inspiration time
·	number of ventilations registered with adequate, too short, too long inspiration time
·	average ventilation flow rate
·	number of ventilations registered with adequate, too short, too long duration
·	number of ventilations registered with airway closed
·	number of cycles registered with too few compressions/ventilations, too many compressions/ventilations, enough compressions/ventilations
·	total hands off time
·	number of cycles registered with correct, too long, much too long, average hands off time
·	total cycles counted

**Figure 3:**
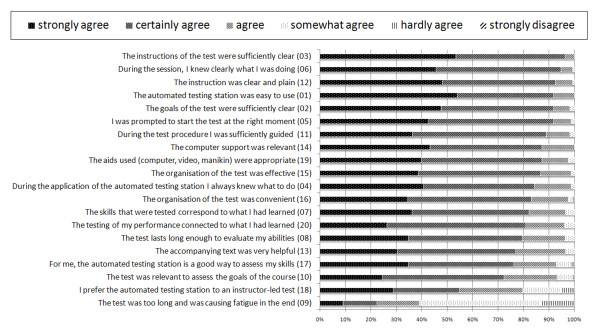
Descriptive results of the questionnaire.

To measure the overall time spent in the testing station (assessment of the efficiency) a research collaborator manually logged the room entrance and exit times during two halve days, resulting in data of a subgroup of 29 students. Six months after completion of the test, all students were asked to complete an online questionnaire regarding their perceptions about being tested in the fully automated testing station (assessment of the effectiveness). In total, 20 items on a six point Likert scale (strongly agree, certainly agree, agree, somewhat agree, hardly agree, strongly disagree) were presented [Figure 3].

### Statistical methods

Results are reported as means with standard deviations. With respect to the questionnaire, descriptive results (based on percentages) are graphically represented.

Students’ responses to the questionnaire were analysed through principal components analysis (PCA) [10]. Since independence of the components was not assumed, an oblique rotation (promax) was used instead of an orthogonal rotation. In order to determine the number of components, parallel analysis (PA) was used. PA is a statistically based method to decide upon the number of components, focusing on the number of components that account for more variance than the components derived from random data, and is more appropriate than using screen plots or the values-greater-than-one rule [11]. Individual loadings of 0.40 or larger were used to identify components. Extracted components were examined and labelled based on the items loading on the specific component. Cronbach’s α was calculated to determine the internal consistency of the items within each component. All statistical analyses were performed using PASW® statistics 18 for Windows (SPSS Inc. Chicago, USA). For the parallel analysis, the SPSS syntax of O’Connor was used [11].

## Results

One hundred and eighty-four students were tested. During testing there was a technical failure (incomplete data registration) in two students and one student performed compressions only. Complete data sets were obtained for the remaining 181 students. According to the automatic time registration of the system, average time on task was three minutes (SD 0.5). Manual timing of the entrance and exit time in the subgroup of 29 students showed an average time of 7.5 minutes (SD 1.4) spent in the testing station.

The questionnaire was completed by 174/184 students (response rate of 94 %). The descriptive results are shown in Figure 3. None of the 20 items received a “strongly disagree” score and only five items (16, 17, 10, 18, and 9) received a “hardly agree” score from a small number of students. Furthermore the graph shows that for the upper 15 questions, more than 80 % of the students either strongly or certainly agreed and that for items 13, 17, and 10 more than 70 % of the students either strongly or certainly agreed. In response to item 18, asking students whether they preferred the automated testing station to an IL test, 55 % of the students either strongly or certainly agreed, 25 % agreed, 15 % somewhat agreed, and 5 % hardly agreed.

### Principal component analysis

We first checked if the data of the 20-item perception questionnaire was suitable for PCA. The Kaiser-Meyer-Olkin value was 0.896, which is above the recommended value of 0.6, and the Bartlett's Test of Sphericity was significant (P < 0.001). Parallel analysis indicated that a two-component structure should be retained. The results of the PCA with promax rotation showed that 50.2 % of the total variance was explained by the two components (respectively 40.6 and 9.6 %). The pattern matrix is presented in Table 2 and shows that 12 items load on the first component, which could be labelled as “instructional organisation”, and seven items load on the second component, which can be considered as “usability”. One item (item nine) was reversed for the PCA, but did not reflect a significant loading (<0.4). Cronbach’s α was 0.92 for the first component and 0.82 for the second component; both larger than 0.80, which is generally seen as a threshold for good internal consistency. Mean scores were 5.3/6 (SD 0.5, range 4.0-6.0) for the first component, indicating that students on average “certainly agreed” to “strongly agreed” about the instructional organisation, and 5.0/6 (SD 0.61, range 3.1-6.0) for the second component, indicating that students on average “certainly agreed” about the usability [Table 2].

**Table 2:** Pattern matrix of the principal components analysis (promax rotation)

**Item**	**Variable**	**Component**	
		**1 Organisation**	**2 Usability**
12	The instruction was clear and plain.	**0.83**	0.02
3	The instructions of the test were sufficiently clear.	**0.78**	0.01
4	During the application of the automated testing station I always knew what to do.	**0.78**	0.05
14	The computer support was relevant.	**0.77**	−0.03
15	The organisation of the test was effective.	**0.76**	−0.06
5	I was prompted to start the test at the right moment.	**0.75**	0.01
2	The goals of the test were sufficiently clear.	**0.71**	−0.03
6	During the session, I knew clearly what I was doing.	**0.71**	0.09
1	The automated testing station was easy to use.	**0.71**	−0.04
16	The organisation of the test was convenient.	**0.69**	0.05
11	During the test procedure I was sufficiently guided.	**0.61**	0.10
13	The accompanying text was very helpful.	**0.57**	−0.09
10	The test was relevant to assess the goals of the course.	−0.18	**0.88**
17	To me, the automated testing station is a good way to assess my skills.	−0.03	**0.85**
18	I prefer the automated testing station to an instructor-led test.	−0.16	**0.77**
20	The testing of my performance connected to what I had learned.	−0.02	**0.76**
19	The aids used (computer, video, manikin) were appropriate.	0.33	**0.52**
7	The skills that were tested correspond to what I had learned.	0.22	**0.48**
8	The test lasted long enough to evaluate my abilities.	0.10	**0.45**
9	The test was too long and was causing fatigue in the end (R).	0.23	−0.10

Note: Values >0.40 are highlighted for ease of interpretation. Item 9 was reversed (R) for the principal components analysis.

## Discussion

We have developed a fully automated testing station to assess BLS skills. An interactive Flash^TM^ module, embedded in commercially available software (Resusci Anne Skills Station^TM^ software), allowed guiding students accurately through the testing procedure without instructor involvement. Although the software contained a timer to indicate the duration of the test, this does not automatically imply that rescuers performed BLS during the full three minutes. By recording the actual time-on-task, we could confirm that average test duration was three minutes. An automated testing station can be used to assess large groups of trainees (i.e. for certificative testing). On a 14 hour per day base, considering an average time of 7.5 minutes per student, eight students could be tested in one hour and in total 112 subjects could be tested in a day. Achieving this number with an instructor would be far more labour- and time-intensive.

Testing stations could also present an added value as an integral part of training, since testing has been shown to yield a powerful effect on retention which may be essential to consolidate newly acquired skills [12]. Adding a test as a final activity in a BLS course seems to have a stronger long-term learning impact as compared to spending an equal amount of time practising the same skills [13-15]. At a theoretical level, the training of continuous retrieval processes seems to account for the “testing effect”. Also, requiring learners to elaborate while retrieving earlier knowledge during testing, has been found to affect long term learning [12,14,15]. Though these assumptions explain the testing effect in relation to declarative knowledge acquisition, the theoretical assumptions also fit the beneficial impact on the acquisition of skills, and tests also invoke retrieval and elaboration of procedural knowledge [14].

The SWOT analysis in Table 3 describes strengths and weaknesses that might affect the achievement of this objective. The automated testing station can also be used in the context of research where there is a need for pre- and post testing of BLS mastery after experimental interventions (i.e. formative testing). The student’s responses to the perception questionnaire indicate that students are positive about the automated testing station. In this respect, the results show that 80 % of the students agreed, certainly agreed or strongly agreed that they prefer an automated testing station to an IL test (item 18). With respect to item nine, the scores were not in line with the other items. Almost 40 % of the students agreed, certainly agreed or strongly agreed that “the test was too long and was causing fatigue in the end”. There may be two reasons for atypical scoring of this item. First, it is the only negatively formulated item in the questionnaire, and students may have overlooked this. Second, it combines two statements, namely “the test was too long” and “the test was causing fatigue in the end”. We also notice the atypical behaviour of this item in the PCA, since it does not reach the threshold loading (higher than 0.4) on the components.

**Table 3:** SWOT analysis of automated BLS skills testing

**Strengths**	**Weaknesses**
· Accessible (24 h/24 h)	· Need for human supervision to supply disposables (wipes and lungs)
· Automated	· Frequent manikin maintenance
· Standardised	· Technical failures (manikin, hardware or software bug, computer problems
· Objective (no instructor bias)	· Hygiene concerns
· Able to achieve adequate testing (effectiveness)	
· within the shortest period of time (efficiency)	
**Opportunities**	**Threats**
· Formative testing of large groups	· Dependency of computer and internet technology
· Certification procedures	· Monopoly of technology and commercial exploitation
· Pre- and post testing in educational interventions	
Acceptance by internet generation	

The PCA resulted in a two-component structure, with one component focusing on the quality of instructional organisation (goals, instructions, assessment and feedback) and the other component focusing on usability. Average scores indicated that students certainly to strongly agreed that the instructional organisation was appropriate and students certainly agreed that the approach was usable. The results of this questionnaire are important for two reasons. First, they show that the automated testing station is functioning properly and is adequately organised. Second, they show that students were positive about the usability of the testing station.

As suggested by Kromann and colleagues, future studies should investigate the intrinsic testing effect and the extrinsic learning effect of formative testing, informing the participant about performance and guiding him towards further skills improvement and mastery [14,15]. These studies could incorporate automated skills testing as a formative assessment procedure in an adaptive learning cycle with repetitive testing [16].

### Limitations

A number of limitations have to be stressed. When discussing the quality of this specific assessment setting, two aspects have to be distinguished. The first aspect is the quality of the assessment process. The second aspect is the quality of the measurement of the performance indicators. This is guaranteed by the intrinsic quality of the manikin sensors and by the use of existing registration software. Maintenance protocols and timely replacement of sensors, valves and springs are imperative to guarantee measurement reliability and validity. In the context of the present study, the students were familiar with training in a SL station and that may have improved the usability of the testing station. However, the automated testing situation and the specific Flash^TM^ module were completely new to the students. Presenting the usability questionnaire six months after testing may have introduced a bias. Further research is needed to confirm these results in terms of non-inferiority compared to IL testing and usability in other student populations.

The software prototype we used only focussed on testing the technical CPR components. Future developments could embed interactive components allowing the trainee to dial a phone number or assessing cardiac arrest by performing the right actions on-screen.

## Conclusions

Automated testing is an effective and efficient method for assessing BLS skills in medicine students and has the potential to innovate traditional resuscitation training. It grounds the scalability of formative assessment and certification procedures without instructor involvement.

## Competing interests

Laerdal Medical (Stavanger, Norway) provided technical support by creating the possibility to replace the existing introduction video of their commercial software package with an interactive Flash^TM^ video. Laerdal Medical also provided the face shields and the RA Skills Station licenses for the duration of the study. The authors have received a grant from the Laerdal Foundation to conduct research in this area. However, Laerdal has taken no part in the design of the study, the analysis of the data or the writing of the current manuscript.

## Authors’ contributions

NM, MV and KM conceived the study, designed the trial, and obtained research funding. NM and KM supervised the conduct of the trial and data collection. NM, MV and KM undertook recruitment of students and managed the data, including quality control. BDW provided statistical advice on http://study design and analysed the data. NM drafted the manuscript, and all authors contributed substantially to its revision. KM takes responsibility for the paper as a whole. All authors read and approved the final manuscript.

## Authors’ information

NICOLAS MPOTOS, MD, MSc, is a resident in emergency medicine and a PhD student at Ghent University Hospital.

BRAM DE WEVER, PhD, is a professor at the Department of Educational Studies, Ghent University.

MARTIN VALCKE, PhD, is a professor and head of the Department of Educational Studies, Ghent University.

KOENRAAD G. MONSIEURS, MD, PhD, is a professor of emergency medicine at the University of Antwerp and at the University of Ghent.

## Pre-publication history

The pre-publication history for this paper can be accessed here:

http://www.biomedcentral.com/1472-6920/12/58/prepub
